# Pre-operative Angiographic Demonstration of Meckel’s Diverticulum with Massive Bleeding

**DOI:** 10.5334/jbr-btr.928

**Published:** 2016-03-02

**Authors:** Ahmet Bas, Deniz Alis, Cesur Samanci, Fethi Ustabasioglu, Selim Bakan, Ayse Cetin

**Affiliations:** 1 Istanbul University, Cerrahpasa Medical Faculty, TR

**Keywords:** Meckel’s diverticulum, Angiography, Embolization, Therapeutic

A 12-year-old child presented to our emergency department with hypovolemic shock due to gastrointestinal bleeding. Gastroduodenoscopy, colonoscopy, and red blood cell nuclear scans were performed, but no definite focal lesions were identified. Emergency visceral angiography was performed.

Contrast medium injection after selective catheterization of the superior mesenteric artery (SMA) showed an abnormal artery branching from the ileal artery and extravasation of the contrast material from that artery. The abnormal artery was thought to be the vitellointestinal artery, which is the feeding artery of Meckel’s diverticulum (Figure 1). After super-selective catheterization of this vessel, therapeutic embolization was performed (Figure 2). Despite this, control angiography at the end of the procedure showed extravasation of the contrast material, so the patient underwent surgery. Exploration revealed Meckel’s diverticulum on the anti-mesenteric side of the ileum (Figure 3a). Diverticulectomy with ileal resection followed by reconstruction with an ileal end-to-end anastomosis was performed. Microscopic examination demonstrated ectopic gastric mucosa accompanying intestinal mucosa in the Meckel’s diverticulum (Figure 3b).

**Figure 1:**
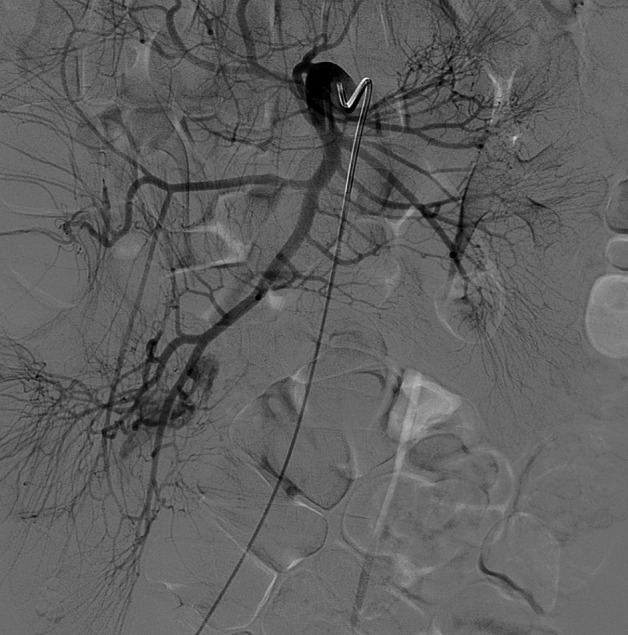
Contrast medium injection after selective catheterization of the SMA shows an abnormal artery branching from the ileal artery and extravasation of contrast material from this abnormal artery.

**Figure 2:**
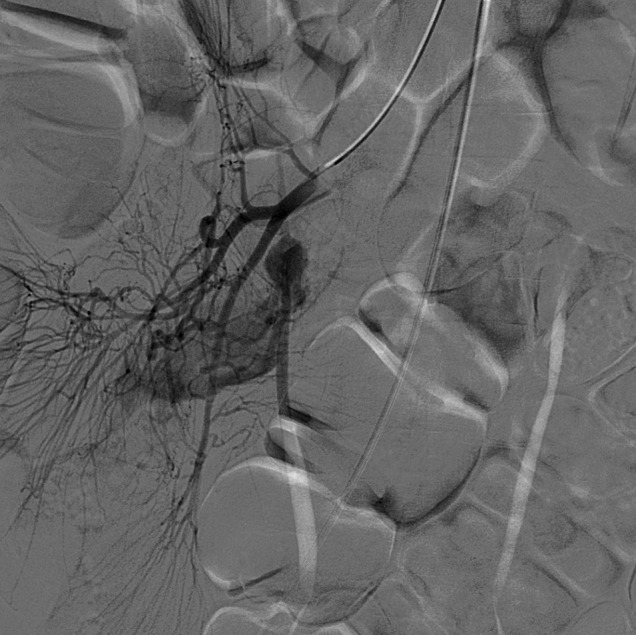
Super-selective catheterization of ileal artery shows persistent vitellointestinal artery and contrast extravasation.

**Figure 3:**
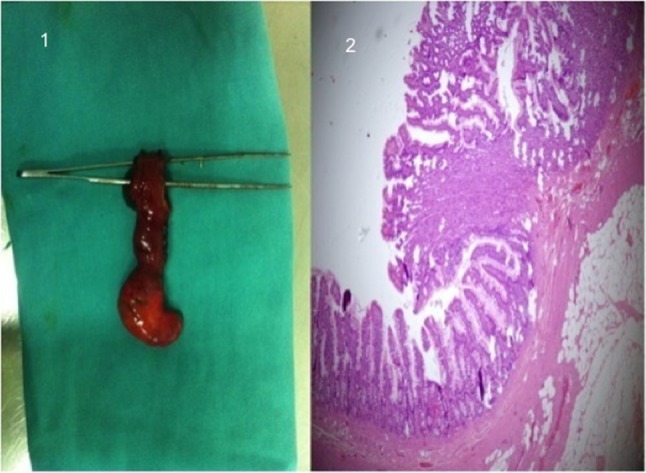
**a)** Removed lesion during surgery. **b)** Microscopic examination demonstrated ectopic gastric mucosa and intestinal mucosa in the Meckel’s diverticulum.

## Comments

Meckel’s diverticulum is one of the most common congenital malformations of the small intestine. It is a true diverticulum, which contains all three layers of the bowel wall [1]. Gastrointestinal bleeding is one of the most common symptoms of Meckel’s diverticula and generally occurs due to erosion of intestinal mucosa secondary to gastric acid secretion from ectopic gastric tissue. In children, severe hemorrhage due to Meckel’s diverticulum is an unexpected entity. Technetium-99m pertechnetate scintigraphy is a feasible technique to localize a site of the intestinal bleeding. However, in emergency conditions, angiography might be a more appropriate tool to localize the origin of the bleeding.

In angiography, hemorrhage appears as a luminal contrast extravasation from the vitellointestinal artery, which is the feeding artery of Meckel’s diverticulum. Furthermore, angiography has the ability to stop massive bleeding by embolization procedures, and it may serve as a transitional step before surgery. Without doubt, definitive treatment of bleeding Meckel’s diverticulum is a surgical excision, and laparoscopy is the technique of choice [2].

In conclusion, although rare, Meckel’s diverticulum may be a reason for massive gastrointestinal bleeding in children, and angiography seems to be a feasible tool for diagnosis and pre-surgical transitional embolization treatment of the massive bleeding.

## Competing Interests

The authors declare that they have no competing interests.
